# The Biomechanical Influence of Step Width on Typical Locomotor Activities: A Systematic Review

**DOI:** 10.1186/s40798-024-00750-4

**Published:** 2024-07-27

**Authors:** Yuan Wang, Qichang Mei, Hanhui Jiang, Karsten Hollander, Pieter Van den Berghe, Justin Fernandez, Yaodong Gu

**Affiliations:** 1 Faculty of Sports Science, Ningbo University, No. 818, Fenghua Rd, Jiangbei District, Ningbo, Zhejiang China; 2 Research Academy of Grand Health, Ningbo University, Ningbo, China; 3 Auckland Bioengineering Institute, The University of Auckland, Auckland, New Zealand; 4 Institute of Interdisciplinary Exercise Science and Sports Medicine, MSH Medical School Hamburg, Hamburg, Germany; 5 Department of Movement and Sports Sciences, Ghent University, Ghent, Belgium; 6 Department of Engineering Science, The University of Auckland, Auckland, New Zealand

**Keywords:** Step Width, Gait Biomechanics, Locomotion, Kinetic Chain, Joints

## Abstract

**Background:**

Step width is a spatial variable in the frontal plane, defined as the mediolateral distance between the heel (forefoot during sprinting) of bilateral feet at initial contact. Variations in step width may impact the lower limb biomechanics. This systematic review aimed to synthesize the published findings to determine the influence of acute changes in step width on locomotion biomechanics and provide implications for injury prevention and enhanced sports performance.

**Methods:**

Literature was identified, selected, and appraised in accordance with the methods of a systematic review. Four electronic databases (Web of Science, MEDLINE via PubMed, Scopus, and ScienceDirect) were searched up until May 2023 with the development of inclusion criteria based on the PICO model. Study quality was assessed using the Downs and Black checklist and the measured parameters were summarized.

**Results:**

Twenty-three articles and 399 participants were included in the systematic review. The average quality score of the 23 studies included was 9.39 (out of 14). Step width changed the kinematics and kinetics in the sagittal, frontal, and transverse planes of the lower limb, such as peak rearfoot eversion angle and moment, peak hip adduction angle and moment, knee flexion moment, peak knee internal rotation angle, as well as knee external rotation moment. Alteration of step width has the potential to change the stability and posture during locomotion, and evidence exists for the immediate biomechanical effects of variations in step width to alter proximal kinematics and cues to impact loading variables.

**Conclusion:**

Short-term changes in step width during walking, running, and sprinting influenced multiple lower extremity biomechanics. Narrower step width may result in poor balance and higher impact loading on the lower extremities during walking and running and may limit an athlete’s sprint performance. Increasing step width may be beneficial for injury rehabilitation, i.e., for patients with patellofemoral pain syndrome, iliotibial band syndrome or tibial bone stress injury. Wider steps increase the supporting base and typically enhance balance control, which in turn could reduce the risks of falling during daily activities. Altering the step width is thus proposed as a simple and non-invasive treatment method in clinical practice.

**Supplementary Information:**

The online version contains supplementary material available at 10.1186/s40798-024-00750-4.

## Key Points

Short-term changes in step width during gait could influence multiple lower extremity biomechanics.

Increasing step width may be beneficial for specific injury rehabilitation.

Wider steps increase the supporting base and typically enhance balance control to reduce the falling risks.

**Graphical abstract:**

**Supplementary Information:**

The online version contains supplementary material available at 10.1186/s40798-024-00750-4.

## Graphical abstract

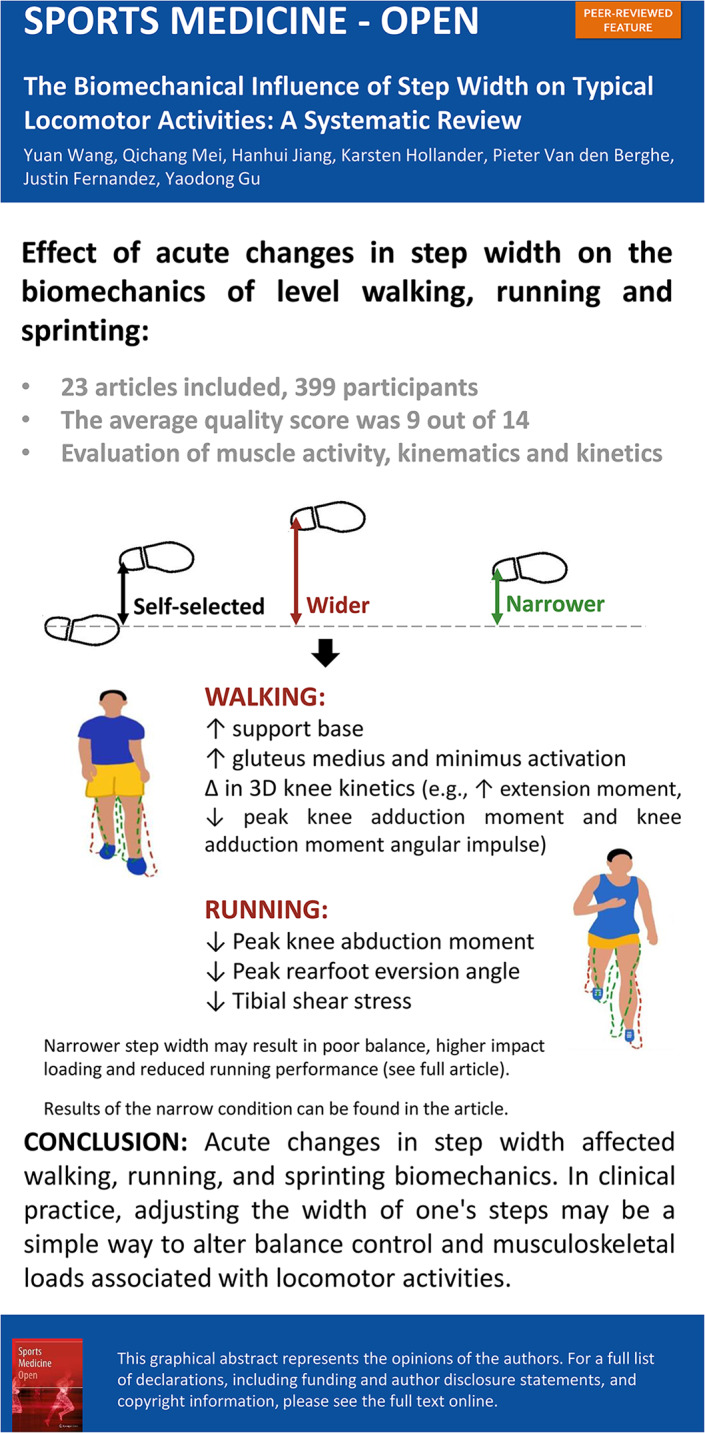

## Background

Human locomotion depends heavily on spatial and temporal factors. Modifying spatial or temporal factors during walking and running can change gait patterns and the associated biomechanics of the lower extremities [1]. Therefore, gait modifications via changes in spatiotemporal parameters could affect several biomechanical factors associated with running-related injuries [2–4]. As reported, optimizing spatiotemporal variables may impact energy expenditure and exercise performance [5, 6], which would produce an optimal economy during walking and running activities [7]. In addition, the change of spatiotemporal variables during walking will also be a challenge to the gait balance at all ages [8, 9]. Among spatiotemporal variables like step frequency (cadence), step length, step width, and contact time, step width is often under-investigated. However, we contend that it may influence the mechanics of lower extremity joints [10].

Step width is a spatial variable in the frontal plane, defined as the mediolateral distance between the heels of bilateral feet at initial contact [11]. Variations in step width and the contributing factors are complex and diverse. As per the findings of previous research, factors such as obesity, sex, age, foot shape and posture, footwear and external conditions have been found to affect the step width [12–24]. Footwear, ground conditions and other external factors would also affect step width [25–29]. For example, obesity could lead to a wider step in all ages [17, 18, 24]. In the context of aging, there is a tendency for step width to increase in the elderly [15, 16]. Functional differences in gait are inherent to sex differences, and females have exhibited a narrower step width compared to males [12, 14, 21]. Pregnancy can also be associated with different step widths in females [13, 19, 20]. Furthermore, Shin et al. [23] found that step width was significantly lower in flatfoot patients compared to symptom-free feet. Previous research reported that shod running widens the step width compared to barefoot running [26, 29]. Additionally, wearing footwear with varying soles and instep flexibility may result in biomechanical variations [28, 29].

Variations in step width may impact the biomechanics in all three planes, and in turn, the function of its constituent components [30]. In the frontal plane, previous research of running reported that a change in step width can alter the rearfoot kinematics [31]. Rearfoot eversion angle peaks and excursion were increased in normal and cross-over running but not during wider step conditions [31]. The kinematics and joint kinetics in the proximal joints (i.e. the knee and hip) are affected by the substantial alterations in step width [11, 32–36]. As the step width narrows in gait, the peak knee abduction moment and impulse decrease [11]. In contrast, the peak knee adduction moment and angular impulse increase [32], along with hip adduction and range of motion (ROM) in hip adduction [34, 35, 37]. The hip adduction moment also increases [36]. In addition to the frontal plane, recent studies have indicated that the step width also influences the biomechanics in the sagittal and transverse planes [33, 37]. Therefore, in our endeavour to better manage overuse injuries, step width is a crucial spatial parameter that warrants exploration in the monitoring and modification of human gait.

The above findings suggest that step width variations and alterations show biomechanical influences. To the best of our knowledge, a systematic review of the actively changing step width in walking, running, and sprinting biomechanics is lacking. Hence, this systematic review aimed to synthesize the published findings to determine the influence of acute changes in step width on locomotion biomechanics, provide implications for injury prevention, and enhance sports performance.

## Methods

The protocol of this systematic review was conducted in accordance with the PRISMA 2020 Guidelines Reporting project for the checklist employed in the current study [38], and was registered at the International Prospective Register of Systematic Reviews (CRD42023445165).

### Search Strategy

We conducted a literature search of the following databases: Web of Science, MEDLINE via PubMed, Scopus, and ScienceDirect. On May 1st of 2023, two researchers (Y.W. and H.J.) independently performed the screening of titles, abstracts, and keywords in these online electronic databases to identify potential studies and searched again on February 1st of 2024, to identify potential new articles between the two search dates. Keywords (MeSH or non-MeSH terms) according to three groups were used in combination with the Boolean indicator “AND” and “OR”. Search terms included ((Step-width OR Step width) AND (run OR walk OR sprint) AND (gait OR biomechanic OR kinetic OR kinematic)). An English language limit was applied. All screened literature was imported into the reference management software (Endnote^®^ version X7, Thomson Reuters, Philadelphia, PA, USA), where duplicate references were removed.

### Eligibility Criteria

The PICO (Patients, Intervention, Comparator, and Outcome) model was used to determine the inclusion and exclusion criteria for the literature in the current systematic review.

#### Inclusion Criteria

We included full-text original research of which the journal paper was peer-reviewed and published in English. This systematic review included the research design in previous studies with the repeated measures experiments, randomized controlled trial (RCT), pre- and post- test design, and pre- and post- test control group.

(1) The population included comprised healthy or pathological adults over the age of 18, without constraints on sex or ethnicity. (2) The study’s intervention targeted substantial differences in step width during walking, running and sprinting. (3) The study had to report an acute comparison of different step widths in level running or walking. (4) The reported outcomes included various biomechanical measures with different step widths, such as spatiotemporal parameters, kinematics, kinetics, electromyography, plantar pressure, etc.

#### Exclusion Criteria

Abstracts, case studies, editorials, reviews, and meta-analyses were excluded. Studies with individuals under the age of 18 were excluded. Studies carried out on stairs or sloping surfaces, without step width intervention and biomechanical outcomes were excluded.

### Data Extraction

After this search process, two reviewers (Y.W. and Q.M.) independently extracted the study characteristics, including the author, date, country, population (sex and age), intervention, motion type (gait pattern), footwear condition, comparisons, outcome (i.e., spatiotemporal parameters, kinematics, kinetics, electromyography, plantar pressure, etc.), results and conclusion. Owing to the lack of comparable data and high-quality studies identified, the meta-analysis was not performed in this systematic review. As a result, the resulting data will be presented descriptively in the tables.

### Quality Assessment

The quality of the associated studies was determined using the modified Downs and Black checklist with 13 of the 27 items from the Downs and Black quality assessment checklist being used following our previous review on walking and running [39–41]. The 13 items in the Quality Assessment Tool (Table 1) might have received the following answers from the reviewers: “Yes,” “No,” or “Cannot Determine.” Any question to which a reviewer responded “Yes” received a score of “1”. Any other response was given a “0”. Thus, the maximum quality score was 14. Zandbergen et al. [42] and Hooper et al. [43] have provided multiple quality labels based on the quality score. A study was considered to be of “Poor” quality if it received a score between 0 and 7, “Fair” quality if receiving a score between 8 and 9, “Good” quality if receiving a score between 10 and 12, and “Excellent” quality if receiving a score of 13 or 14. In this case, the tool is appropriate because it is suitable for all types of quantitative research designs [44]. Each study’s quality was evaluated separately by two researchers (Y.W. and H.J.), and the quality assessment disagreements were addressed until a consensus was reached. If consensus was not reached, a third reviewer (Q.M.) made the final decision.

**Table 1:** Quality assessment for included studies

Study	①	②	③	④	⑤	⑥	⑦	⑧	⑨	⑩	⑪	⑫	⑬	Total	Quality
Pohl et al. [31]	1	1	1	1	1	0	1	1	0	0	1	1	0	9	Fair
Meardon et al. [37]	1	1	1	1	1	0	1	1	1	0	1	1	0	10	Good
Singer et al. [45]	1	1	1	1	1	0	1	1	1	0	1	1	0	10	Good
Young et al. [8]	1	1	0	1	1	0	1	0	1	0	1	1	0	8	Fair
Young et al. [46]	1	1	0	1	1	0	1	0	1	0	1	1	0	8	Fair
Nagano et al. [47]	1	1	1	1	1	0	1	1	1	0	1	1	0	10	Good
Brindle et al. [11]	1	1	1	1	1	0	1	1	1	1	1	1	0	12	Good
Meardon et al. [48]	1	1	1	1	1	0	1	1	1	0	1	1	0	10	Good
Kubinski et al. [49]	1	1	1	1	1	0	1	1	1	0	1	1	0	10	Good
Arvin et al. [9]	1	1	1	1	1	1	1	0	1	0	1	1	0	10	Good
Favre et al. [32]	1	1	1	0	1	0	1	1	0	0	1	0	0	7	Poor
Bajelan et al. [50]	1	1	0	1	1	0	0	1	1	0	1	1	0	8	Fair
Bennett et al. [33]	1	1	0	1	1	1	1	1	1	1	1	0	0	10	Good
Maharaj et al. [30]	1	1	1	1	1	1	0	1	1	0	1	1	0	10	Good
Sandamas et al. [51]	1	1	1	1	1	1	1	1	1	0	1	1	0	11	Good
Kikel et al. [34]	1	0	0	1	1	0	1	1	1	1	1	1	0	9	Fair
Sample et al. [35]	1	1	0	1	1	1	1	0	1	1	1	1	0	10	Good
Rawal et al. [52]	1	1	1	1	1	0	1	1	1	0	1	1	0	10	Good
Shih et al. [53]	1	1	1	1	1	1	1	1	1	0	1	1	0	11	Good
Stief et al. [36]	1	1	1	1	1	1	1	1	1	0	1	1	0	11	Good
Wang et al. [54]	1	1	0	1	1	0	0	0	0	0	1	1	0	6	Poor
Alizadehsaravi et al. [55]	1	1	1	0	1	0	1	1	0	0	1	0	0	7	Poor
Magnani et al. [56]	1	1	1	1	1	1	0	1	1	0	1	0	0	9	Fair
Criterion fulfilled in % of studies	100%	96%	70%	91%	100%	35%	83%	78%	83%	20%	100%	82%	0%	/	/

① Is the hypothesis/aim/objective of the study clearly described? ② Are the main outcomes to be measured clearly described in the Introduction or Methods section? ③ Are the characteristics of the patients included in the study clearly described? ④ Are the interventions of interest clearly described? ⑤ Are the main findings of the study clearly described? ⑥ Does the study provide estimates of the random variability in the data for the main outcomes? ⑦ Have actual probability values been reported (e.g. 0.035 rather than < 0.05) for the main outcomes except where the probability value is less than 0.001? ⑧ Were the subjects asked to participate in the study representative of the entire population from which they were recruited? ⑨ Were the staff, places, and facilities where the patients were treated, representative of the treatment the majority of patients receive? ⑩ If any of the results of the study were based on “data dredging”, was this made clear? ⑪ Were the statistical tests used to assess the main outcomes appropriate? ⑫ Were the main outcome measures used accurate (valid and reliable)? ⑬ Did the study have sufficient power to detect a clinically important event where the probability value for a difference being due to chance is less than 5%?

## Results

### Search Results

**Fig. 1:**
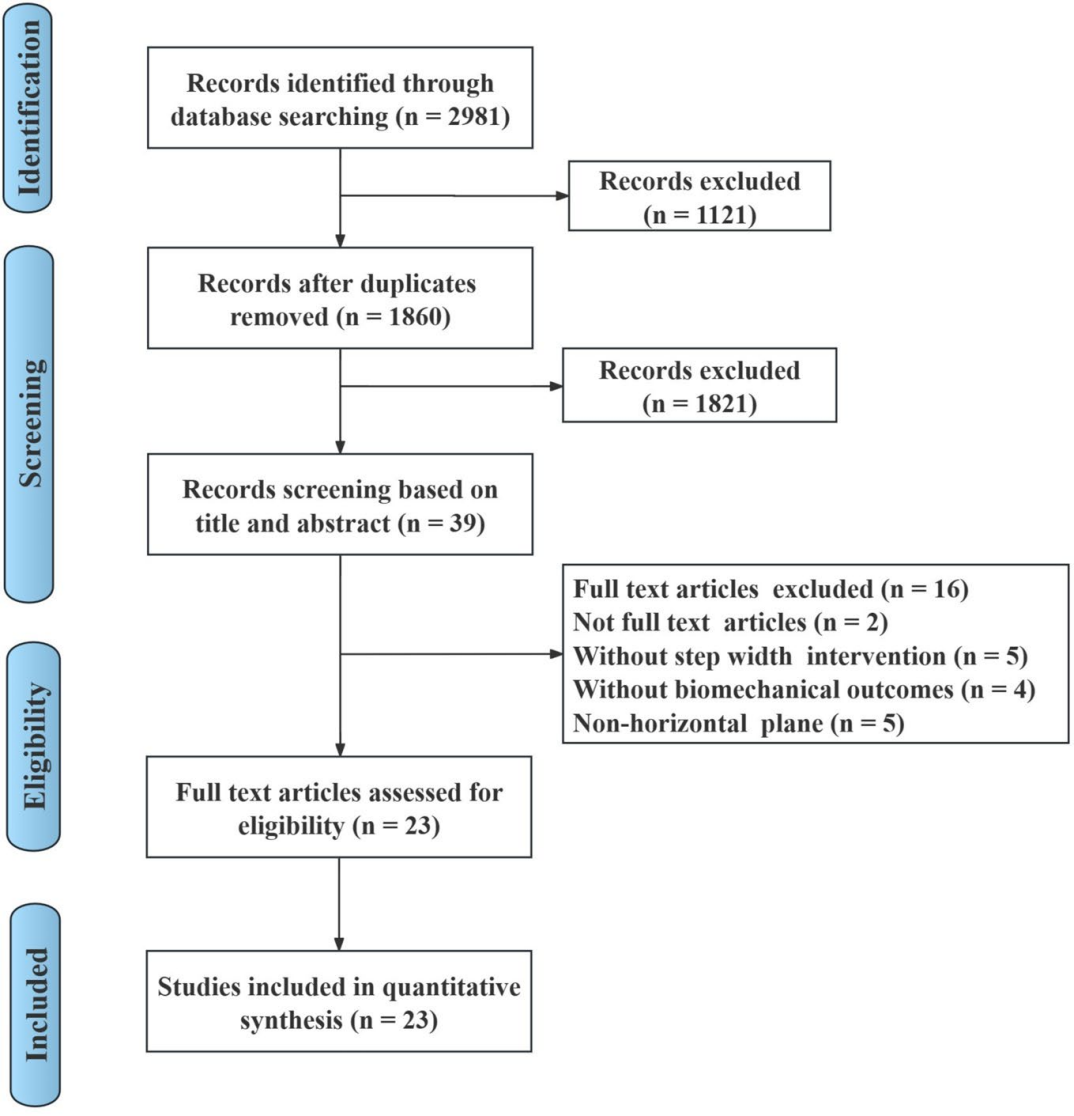
Flow diagram of the systematic review study selection process

The primary search resulted in 2981 articles from the electronic databases. After deleting 1121 duplicate articles based on the title and abstract, 1860 articles were examined and 1821 were removed. The full texts of the remaining 39 articles were examined. Twenty-three articles met the inclusion criteria and were ultimately included in the systematic review. The flow diagram of this systematic review is shown in Fig. 1.

### Quality Assessment Results

The average quality score of the 23 articles included was 9.39 out of 14. Among these articles, 14 studies were ranked as “Good” with scores ranging from 10 to 12 [9, 11, 30, 33, 35–37, 45–53]. Six studies were ranked as “Fair” with scores of 8 or 9 [8, 31, 34, 46–56]. and 3 studies were considered as “Poor” with scores ranging from 0 to 7 [32, 54, 55]. None of the included studies reached the level of “Excellent”. Table 1 lists all the scores from the quality evaluation.

### Study Characteristics

The current review classified 23 studies into 3 categories according to the motion type of research, specifically the walking activity (Table 2), running activity (Table 3) and sprinting activity (Table 4). Each table provided a full overview of the characteristics of the research subjects included in this systematic review. The 23 studies included 399 participants. There was a total of 213 males and 148 females included in 22 studies, and only one study did not report the sex [33]. The mean age of the adult population was 27.32 (± 10.53) years. Two articles only included males [45, 47], and in one of them Nagano et al. [47] included young as well as old participants.

**Table 2:** Walking activity

Study	Country	Subject description	Intervention	Motion type	Footwear condition	Comparisons	Outcome	Result	Conclusion
Singer et al. [45]	Canada	10 healthy young male participants (age 24.1 (2.9) years)	Preferred step length/width (PREF1); Increased step length (ML preferred) (AP); Increased step width (ML). Increased AP step length and step width (AP&ML).	Walking	-	PREF1 VS AP VS ML VS AP&ML	Kinematic Analysis:	Overshoots of the final COM position were most prevalent, occurring in 77% (AP) and 68% (ML) of all trials in the PREF1 condition.	Trials with increasing step width showed larger levels of incongruity and overshoot in the frontal plane.
Young et al. [8]	USA	13 young healthy adults (7 males, 6 females; age, 18–35)	Walk with narrower (NA) steps than normal (NO).	Walking	-	WI VS NA VS NO.	Margin of stability (MOS), MOS variability,	Wider steps caused MOSap decreased, MOSml increased, and increased MOSap and MOSml variability.	Gait training programs advise making short-term modifications to step characteristics to increase stability.
Young et al. [46]	USA	14 young healthy adults (7 males, 7 females; age 18–35)	Walk with wider (WI) and narrower (NA) steps than normal (NO).	Walking	-	WI VS NA VS NO	Space-time Analysis:	Walking with wide steps increased SW variability, decreased SL variability, and made participants’ C7 marker movements exhibit increased short-term local instability in all directions of motion.	Short-term intentional adjustments in SW did affect the stability of the trunk’s local and orbital movements.
Nagano et al. [47]	Australia	30 young (18-35 yrs.) and 26 older male adults (> 60 yrs.) with identical physical characteristics, including height (young: 1.77 ± 0.06 m, older: 1.74 ± 0.07 m) and mass (young: 75.7 ± 3.5 kg, older: 76.7 ± 7.9 kg).	Preferred, narrow and wide step width. Narrow and wide walking conditions were ±50%	Walking	-	Preferred VS narrow VS wide step width.	Kinematic Analysis:	Minimum lateral margin and COM variability increased in narrow and wide step width conditions. Step width variability was reduced in increased and decreased step width.	Despite having constant foot control under settings of changed width, there is less consistent medio-lateral COM control.
Kubinski et al. [49]	USA	14 adults (10 female, 4 male; age = 24±2 years; mass = 64.8±11.2 kg; leg length = 0.8 ±0.06 m).	Narrow study took narrow steps, such as walking with foot straight in front of one another. Medium trial walked with a normal step width (not too narrow or too broad), but to maintain this step width throughout the gait. Wide experiment took the broadest steps to sustain during the walking session.	Walking	-	Narrow VS Normal VS Medium VS Wide.	EMG Analysis:	Gluteus medius activity increasing with wider steps during both prescribed and normal walking.	Adding step width increases the need for strong stance phase hip abductor contractions among healthy controls.
Stief et al. [36]	Germany	20 healthy individuals (24.0 ± 2.5 years of age)	Self-selected step width	Walking	Barefoot	Habitual SW condition VS other 3 conditions	Kinematic Analysis:	Increased SW during gait reduced the first and second peak knee and hip adduction moments	Increased SW may be an adequate, noninvasive option for the treatment of patients with hip osteoarthritis.
Bajelan et al. [50]	Australia	4 healthy young male adults (23.0 ± 2.2 yrs) with stature and body mass; 1.73 cm-60Kg, 1.68 cm-60Kg, 1.72 cm-65Kg and 1.78 cm-85Kg respectively.	Wide walking conditions were +50% relative to step width in unconstrained walking	Walking	-	Normal VS Wide step width.	Space-time Analysis:	Step length increases with step width. Walking with wide steps make Gluteus Minimus muscles more active.	Wide step walking was shown to employ more abductors and use less adductor forces.
Arvin et al. [9]	The Netherlands	18 healthy, community-dwelling older adults (10 females; mean age 73, SD 4 years; height 172, SD 10 cm; mass 63, SD 6 kg) and 14 young adults (9 females; mean age 23, SD 3 years; height 174, SD 10 cm; mass 66, SD 10 kg).	Preferred SW (PSW) or at 50% of their preferred SW (NSW).	Walking	-	PSW VS NSW	Kinematic Analysis:	Smaller ML-MOS, ML-COM displacement, ML-COM velocity and ML-xCOM amplitude in the NSW condition than in PSW. COM velocity variability was higher in NSW than in PSW.	Particularly in elderly persons, narrowing step width may make it difficult to maintain ML-balance control and result in a less stable gait.
Kikel et al. [34]	USA	10 subjects (5 females, 5 males; body mass = 68.5 ± 11.5 kg; age = 25.4 ± 1.9 years).	Walk with varying step width conditions (normal, narrow, wide). For the narrow step width condition, subjects were instructed to walk with feet falling along a straight line. For the wide step widths, subjects walked with feet outside of two strings placed on the treadmill.	Walking	-	Normal VS Narrow VS Wide step width	Kinematic Analysis:	Narrow step width increased average hip adduction and displayed a larger hip adduction range of motion than either normal or wide step widths.	The evolution of valgus knees and narrow steps in humans may be decoupled from the evolution of the human-like pattern of pelvic list
Sample et al. [35]	USA	13 adult participants (7 males and 6 females), 7 healthy-weight (6 females and 1 male; age: 23.29 [2.60] y; body mass index [BMI]: 21.76 [1.92]), and 6 obese (6 males; age: 25.33 [2.81] y; BMI: 32.20 [2.78])	For the wide step-width conditions, 13% of the participant’s leg length was added to the participant’s preferred step-width.	Walking	Unified laboratory footwear	Preferred SW	Kinematic Analysis:	As step-width increased, medial GRF and knee extension moment were increased but peak knee adduction angle was reduced.	Increasing step-width may be a useful strategy for increasing knee extension moment in a healthy and young population.
Alizadehsaravi et al. [55]	The Netherlands	22 older (72.6 ± 4.2 years old; mean ± SD, 11 females) healthy volunteers	A narrow-base walking paradigm	Walking	-	narrow-base VS normal walking	Kinematic Analysis:	Compared to normal walking, the CoM displacement variability in narrow-base walking was greater, improved stability, activation duration was shorter for synergies associated with dominant leg weight acceptance and non-dominant leg stance, and longer for the synergy associated with non-dominant heel-strike.	It is possible to interpret the synergistic adaptations to narrow-base walking as being connected to more careful weight transfer to the new stance leg and improved control of COM movement in the stance phase.
Rawal et al. [52]	Canada	25 participants (mean age: 23(3) years; mean height: 1.72(0.10) m., mean body mass: 64.4(11.5) kg; BMI: 21.7 (2.58) kg/m2; 52% female).	Self-selected speed and foot-	Walking	-	Normal-walking	Kinematic Analysis:	Narrow-base walking reduced stability as evidenced by reduced MoSML and reduced the peak GRFnet eccentricity.	Proactive mechanisms may predominantly regulate mediolateral stability during walking.
Shih et al. [53]	USA	20 healthy young adults (14 females, 6 males; 26.25 ± 3.31 years; 165.54 ± 9.93 cm; 61.39 ± 12.71 kg; BMI = 22.21 ± 2.84 kg/m2).	Five different step widths, including 0.33, 0.67, 1, 1.33, and 1.67 × preferred step width (PSW).	Walking	-	0.33 VS 0.67 VS 1 VS 1.33 VS 1.67 PSW	Kinematic Analysis:	Amplitude of mediolateral CoM deviation scaled with step width. Step width changes also affected Trunk kinematics in all three planes. Both peak longissimus activation and bilateral longissimus co-activation increased at narrower step widths, while at greater widths, bilateral co-activation decreased more markedly than peak activation.	Both wider and narrower step widths place unique demands on trunk control.
Bennett et al. [33]	USA	38 healthy individuals with varus, neutral, and valgus knee alignment	Self-selected normal, increased toe-in (TI) targeted at 10° with self-selected step width, and increased toe-in targeted at 10° with a step width of 26% leg length (TIW) gaits	walking	Unified laboratory footwear	Normal VS TI VS TIW	Kinetics Analysis:	Reduced peak knee adduction moment and impulse in TIW compared to Normal gait. Increased peak knee flexion and external rotation moments in TIW compared to normal gait.	The TIW gait is recommended to reduce peak knee adduction moments and knee adduction impulses in people with varus alignment.
Magnani et al. [56]	Brazil	14 healthy participants (5 female/9 male; mean age 28.42 ± 2.87 years)	Step width conditions (control, narrow-base, wide-base), for narrow-base walking, participants were asked to adopt a smaller base of support (smaller than hip width); for wide-base walking, participants were asked to increase the base of support adopting step width greater than hip width.	Walking	Optional footwear	Control VS narrow-base VS wide-base	Kinematic Analysis:	CoM variability was smaller in normal than in narrow-base walking. Step width variability was smaller in the control condition than narrow-base walking.	Walking in narrow-base condition was more stable.
							Center of mass (CoM)		
							Space-time Analysis:		
							step width variability		
Favre et al. [32]	Switzerland	10 healthy subjects (5 male, 25.2± 2.9 years, 1.71±0.06 m, 63.7±5.9 kg) without history of lower limb injury.	step width (decreased, normal, and increased)	Walking	-	Narrow step width VS preferred step width VS wide step width	Kinematic Analysis:	Wider step width reduced first knee adduction moment, peak and knee adduction moment angular impulse.	Increasing trunk sway, increasing step width, and toeing-in are three gait modifications that can be combined to reduce KAM variables related to knee osteoarthritis.
Maharaj et al. [30]	Australia	12 individuals (7 female; height: 1.73 ± 0.08 m; body mass: 67 ± 8 kg; age: 23 ± 3 yrs.)	Step widths included each subject’s preferred width	Walking	Barefoot	Preferred width VS 0.1 L VS 0.2 L VS 0.3 L VS 0.4 L step width	Kinematic Analysis:	Walking at step widths greater than preferred reduced peak STJ moments at initial contact and propulsion which subsequently reduced the negative and positive work performed at the STJ. Increased knee and hip energy absorption during initial contact.	Increase in step width likely to be beneficial in the prevention and treatment of STJ injuries.

**Table 3:** Running activity

Study	Country	Subject description	Intervention	Motion type	Footwear condition	Comparisons	Outcome	Result	Conclusion
Meardon et al. [37]	USA	8 males and 7 females experienced runners (age: 23.7 ± 5.36 years; height: 174.0 ± 7.5 cm; mass: 70.3 ± 9.19 kg)	Narrow, preferred, and wide step widths.	Running	Optional footwear	Narrow step width VS preferred step width VS wide step width	Kinematic Analysis:	Greater hip adduction in the narrow condition than the preferred condition; preferred condition greater than the wide condition. Greater knee internal rotation in the narrow condition than the preferred and wide conditions. Increased ITB strain and strain rate with narrower the step width in a linear trend.	Wider step width may be beneficial
Meardon et al. [48]	USA	8 males and 7 females, 23.7±5.4 years,	Narrow, preferred, and wide step width.	Running	-	Narrow step width VS preferred step width VS wide step width	Kinematic Analysis:	Increasing step width reduced anterior tension, posterior compression, and medial compression of the tibia, linearly reduced shear stress at all sites.	Prevention of sports injuries should consider the characteristics of stride width
Brindle et al. [11]	USA	30 healthy adults, fifteen men and fifteen women, 18–35 years of age.	20% of leg length and the narrow step width condition was 0%	Running	Unified laboratory footwear	Preferred VS wide vs. narrow step width.	Knee joint, Hip joint and Rearfoot	Peak rearfoot inversion moment increased.	Biomechanics of the rearfoot, hip and knee joints are affected by step width.
Pohl et al. [31]	UK	12 subjects (6 males, 6 females; mean age (SD), 29.9 (4.9) years; body mass, 61.2 (15.1) kg; and height,	A cross-over condition (Xover); a wide condition (Wide); and a normal condition (Norm).	Jogging	Barefoot	Xover VS Wide VS Norm	Kinematic Analysis:	Peak rearfoot eversion of Xover was greater than Wide and normal condition.	Rearfoot frontal plane motion had a significant coupling with transverse shank rotation, forefoot sagittal plane motion, and forefoot transverse plane motion.
		171.2 (9.5) cm)							

**Table 4:** Sprinting activity

Study	Country	Subject description	Intervention	Motion type	Footwear condition	Comparisons	Outcome	Result	Conclusion
Sandamas et al. [51]	Sweden	10 competitive sprinters (8 male and 2 female) (mean ± SD: age, 23 ± 6 years, height 1.77 ± 0.10 m, mass 72.7 ± 13.6 kg,	skating and narrow trials when the 1st step width	Sprinting	-	Skating VS narrow	Kinematic Analysis:	Narrow steps reduced medial block and medial 1st stance impulses, 1st stance anterior toe-off velocity and mediolateral motion of the CoM, medially directed forces and mediolateral motion of the COM.	Reducing step width did not lead to any improvement in performance, skating style was shown to have a greater propulsive impulse during the 1st stance.
		personal best: men 11.03 ± 0.36 s, women 11.6 ± 0.45 s)							
Wang et al. [54]	Sweden	4 (2 male and 2 female) competitive sprinters (mean ± SD: height, 1.75 ± 0.10 m; mass,70.25 ± 14.04 kg)	Sprint	Sprinting	-	widest step width VS narrowest step width	Kinetics Analysis:	Narrow trials reduced COM propulsion and particularly support.	Narrow steps might inhibit athletes’ performance in sprint

All 23 articles included in this systematic review compared the biomechanical differences from altered step widths in walking, running or sprinting activities. Of the 23 studies, 17 were conducted during walking, 6 were conducted during running and 2 were conducted during sprinting. Fourteen of the 23 studies compared a narrow step width, the preferred (habitual) step width and a wide step width. Four of the 23 studies compared the preferred step width to a greater width. Finally, five of the 23 studies compared the preferred step width to a narrower width.

### Biomechanical Effects of Altering Step Width

All 23 studies included in this systematic review compared the biomechanical differences from altered step widths.

#### Spatiotemporal Changes Following a Change in Step Width

Three of the included papers assessed spatiotemporal parameters after altering step width [46, 47, 56]. Walking with wider and narrower step widths significantly increased step width variability compared to control condition [46, 47, 56]. Both narrow and wide walk steps were associated with increased mean stride time and decrease in stride time variability [46]. In addition, narrow steps increased average step length and step length variability whereas wide steps decreased average step length and conversely increased the step length variability [46].

#### Kinematic Changes Following a Change in Step Width

Thirteen of the included articles assessed kinematic parameters when altering step width. A change in step width led to changes of trunk kinematics [53], and also to change in lower extremity joint kinematics [11, 31, 34, 35, 37].

During running, narrow step width increased the peak rearfoot eversion [31], but wider step width condition reduced peak rearfoot eversion angles compared with the narrow and preferred conditions [11, 31]. Knee internal rotation varied depending on step width and was higher in the narrow steps than in the normal condition; however, no statistically significant changes were discovered between the preferred and wide conditions [11, 37]. In the hip joint, a wide step width reduced average hip adduction angle [11, 37].

During walking, the kinematics of the hip joint showed the same changes as running when changing the step width but displayed a smaller range of motion of hip adduction than either narrow or normal step width conditions in locomotion biomechanics [34, 35].

Center of mass (COM) position was also associated with changes in step width during walking. Variability of COM and variability of COM velocity increased in decreased step width conditions to narrow-base condition [9, 47, 55, 56]. Arvin et al. also discovered that increasing the step width resulted in more COM variability than the preferred step width [9]. Mediolateral (ML)-COM kinematics deviation scaled with step width, less ML-COM displacement in the narrow condition [8, 45, 51, 53], as well as narrow step width condition, presented with lower ML-COM velocity [9]. Additionally, the margin of stability (MOS) from anterior–posterior (AP-MOS) and mediolateral (ML-MOS) directions were also affected by the step width conditions [8, 9, 52]. Walking with narrow steps decreased ML-MOS significantly, while walking with wide steps increased AP-MOS and decreased ML-MOS significantly [8, 52]. Further, Young et al. (2012) found that wide step width was linked to increased AP-MOS and ML-MOS variability [8]. However, Arvin et al. failed to observe a difference in the ML-MOS’s variability with different step widths [9]. In addition, during sprinting, COM can also be affected by step width; narrower steps reduce COM propulsion and particularly support [54].

#### Kinetic Changes Following a Change in Step Width

Ten of the included papers assessed kinetics parameters after altering step width. Previous studies found that step width could alter the kinetics of the hip joint, knee joint, ankle, subtalar joint (STJ) and rearfoot [11, 30, 32, 33, 36].

In running, when the step width changed from wide to narrow, the peak rearfoot inversion moment increased [11]. The narrow step-width condition had a larger peak rearfoot inversion moment than the wide step width condition, while the preferred step width had a larger peak rearfoot inversion moment than the wide steps [11]. Peak knee abduction moment and impulse were smaller in the preferred and wider step conditions compared to the narrow step condition during running, and were also smaller in the wider step condition compared to the preferred step width [11].

In walking, step width had an impact on the knee kinetics in all three planes [11, 30, 32, 33, 35, 36]. In the sagittal plane, peak knee flexion moments increased in wider step width with toe-in compared to normal gait [33], and knee extension moment was larger with increased step width [35]. In the frontal plane, wider step resulted in decreased peak knee adduction moment and knee adduction moment angular impulse [32, 33, 36]. In the transversal plane, peak knee external rotation moments were greater in larger step width with toe-in compared to normal gait [33]. In the hip joint, increased step width during walking reduced peak hip adduction moment [36]. Furthermore, the tibia was mainly loaded when the step width was narrower, and iliotibial band strain and strain rate showed a linear increasing trend as the step width narrowed [37, 48].

Peak STJ moments and propulsion were lowered when walking at larger step widths than preferred during initial contact, but knee and hip energy absorption increased at the initial contact [30].

As step width increases during walking, the medial ground reaction force (GRF) and eccentricity of the net GRF were increased [35, 52]. During sprinting, the vertical and forward GRF peaks were higher in the natural control condition compared to the narrow-width conditions [54].

#### EMG Changes Following a Change in Step Width

Five of the included articles assessed muscle activation via EMG following an alteration in step width. Lower extremity muscle activity was susceptible to step width alterations. More specifically, the activities of gluteus medius and gluteus minimus increased as step width increased in walking [49, 50]. On the contrary, peak longissimus activation and bilateral longissimus co-activation both decreased at wider step widths [53]. Furthermore, the peak activation duration in narrow walking conditions associated with the dominant leg stance was delayed compared to normal walking, but occurred earlier in synergies associated with non-dominant leg stance [55].

Under the narrow condition, when the first step of the sprint was taken, the soleus, gastrocnemius, rectus femoris, vasti, gluteus maximus, gluteus medius, biceps femoris, and adductors contributed less to propulsion and support [54].

## Discussion

The aim of the current study was to systematically synthesize literature that investigated the gait changes induced by step width alterations from a biomechanical perspective. In the included 23 articles, a total of 399 healthy adults performed walking (*n* = 17/23), running (*n* = 4/23) or sprinting (*n* = 2/23) under different step width conditions and multiple biomechanical aspects were analyzed, including the joint kinematics, joint kinetics, spatiotemporal parameters, and EMG (muscle activities). The average score of the methodological quality was 9.39 out of 14, which indicates a “Fair” level of quality according to the Downs and Black quality assessment.

A major finding from this review was that step width alteration affected the joint kinematics and kinetics in all three planes of movement, such as peak rearfoot eversion angle and moment [11, 31], peak hip adduction angle and moment [11, 34–37], knee flexion moment [33], peak knee internal rotation angle as well as knee external rotation moment [11, 33, 37]. Another finding was that step width alterations do affect the stability and posture during walking, running and sprinting, and are expressed as the transformation of COM and MOS locations in anterior–posterior and mediolateral directions [8, 9, 47, 51, 53, 55, 56]. It was specifically reported that the muscle activity, GRF, spatiotemporal parameters, tibial stress, iliotibial band strain, and strain rate would be affected by different step width conditions [35, 37, 47–49, 52–55]. The study outcomes will be discussed in three categories, covering daily activities, clinical treatment, and athletic training.

### Daily Activities

During daily activities, the strategies to prevent sports injuries and falls, especially among the elderly, have long been a focus of particular concern. One primary objective of human daily activity is to maintain stability and prevent falling. Adjusting the posture during gait occurs to actively control the COM variability and maintain trunk stability; further experiments showed that increasing step width increased trunk lean and, as a result, increased COM displacement and reduced knee abduction moment across the stance phase [57–60]. Narrow step width that reduced the support base during walking increased the need for active postural control and presented a greater challenge for stability [61, 62]. This finding also agreed with observations from the current systematic review, showing that the COM displacement variability increased in decreased step width and narrow steps [47, 55, 56]. Similarly, this finding was consistent with studies of MOS that found that walking with narrower steps exhibited poorer stability, as evidenced by reduced ML-MOS [9, 52], when paired with greater ML-MOS variability [8, 9], in particular in the elderly [9]. Therefore, narrow step widths should be avoided as much as possible to reduce the risk of balance loss and falling.

### Clinical Treatment

Under the scenarios of clinical treatment, gait retraining for osteoarthritis (OA) individuals with an increased step width may be a suitable, noninvasive therapeutic option [32, 33, 36]. Reduced initial peak knee adduction moment and knee adduction moment angular impulse during gait were the results of wider step width [32]. The finding that gait adjustment could improve knee biomechanics related to knee OA was consistent with reports by Bennett et al. [33], investigating that peak knee adduction moment and impulse decreased in a wider step width with toe-in compared to normal walking. Furthermore, improvements also occurred in the hip joint as increased step width during gait reduced the peak hip adduction moment, making an effective compensatory mechanism to relieve hip OA as well as loading in the hip joint [36].

Altering step width during level walking and ascending and descending stairs would change lower limb biomechanics [63–66]. Although the stairs-related gait studies were not included in this systematic review, non-horizontal movements of stairs played important roles in improving daily activities for patients with knee OA. Previous investigations demonstrated that increased step width while descending stairs resulted in lower peak knee adduction angles and moments, which may suggest that lowering medial compartment knee loads might thus potentially reduce knee pain [63, 67]. However, while analyzing OA patients, the findings were the opposite, i.e. increased step width could not decrease internal knee abduction moments peak or knee pain [64]. When participants ascended the stairs, Paquette et al. [65] and Yocum et al. [66] found that increasing step width reduced knee extension and abduction ROM, peak knee abduction moments, knee abduction moment impulse, and GRF in the frontal plane. Consequently, increased step width would probably be an effective and easy gait modification for reducing joint loads and arthralgia in both OA individuals and healthy persons [32, 33, 36, 63–66]. This finding may have positive clinical significance to prevent disease progression.

Previous studies reported that symptomatic runners, such as those who suffer from patellofemoral pain (PFP) and iliotibial band syndrome (ITBS), exhibited different lower limb biomechanics [68–74]. In the prospective studies, peak hip adduction angles and knee internal rotation were found to be higher in runners who later suffered from ITBS [69, 72]. Knee internal abduction moment and impulse were also larger in runners with PFP [68, 73]. Nevertheless, the connection between peak hip adduction angle and PFP has been disputed with no consensus achieved [70, 71, 73, 74]. As for the variations in step width, it was found that the maximum knee internal rotation angle was greater during narrow step width compared to preferred step width [11, 37]. Consistent with the above literature, it was found that participants with narrower steps demonstrated that narrow step width in gait showed similar lower limb biomechanics in patients with ITBS [11, 34, 35, 37]. Specifically, the narrower step width displayed a higher average hip adduction angle compared to both normal and narrow step widths [34, 35]. On the other hand, both PFP runners and participants with narrow steps in gait showed greater peak knee abduction moment and impulse [11, 33, 35]. As a result, the biomechanics of asymptomatic runners were comparable to runners with lower limb injuries while running with small step widths. Additionally, runners with ITBS showed greater peak rearfoot inversion moments compared to asymptomatic runners [72]. Brindle et al. identified that the rearfoot inversion moment peak was reduced as step width increased from narrow to preferred and wide [11]. Based on these findings, while running with a wider step and avoiding narrower steps, the biomechanical parameters changed in the opposite way to runners with lower extremity disorders. Hence, the injury risks of runners with inappropriate frontal plane biomechanics may be reduced by raising the preferred step width.

OA patients and patients suffering from ITBS and tibial injuries may benefit from altering step width during running gait [30, 37, 48]. In addition to indirectly improving biomechanical parameters associated with ITBS [11, 34, 35, 37], the increased step width also directly and linearly reduced iliotibial band (ITB) strain and ITB strain rate during running [37]. Wider steps may be advantageous for the treatment and prevention of ITBS. A linearly increased step width could decrease tension on the tibia surface [48] and reduce the STJ moments during stance, thus reducing the work for the musculoskeletal system. The tibialis posterior tendon may experience less strain and stress as a result of the reduced STJ loading [30]. In summary, a wider step width may benefit runners with major general running-related musculoskeletal injuries (i.e. posterior tibial tendinopathy and tibial bone stress injuries) and runners who participated in ultra-marathon races with the most common running-related musculoskeletal injury (ITBS).

### Sports Training

In sports training, especially during sprinting, the competition results and athletic performance may be affected by restricted starting step width and position [51, 54]. Compared to the natural sprint style, a restricted step width would decrease mediolateral propulsive impulse and first-step stance toe-off anterior velocity [51]. The natural sprint style in the first step exhibited greater mediolateral motion of the CoM, representing larger lateral external forces during the block and initial stance phases [51]. Furthermore, competitive sprinters with limited step width showed reduced extremity muscle contribution to propulsion and support, suggesting that narrower steps may suppress the muscles across the ankle and knee for maximal performance at sprinting start [54]. These findings suggested that the development of driving force during the first stance of the acceleration phase may be best achieved with a wider step width.

However, prior research demonstrated unequivocally that increased step width would raise metabolic expenditure during running and walking [5, 75, 76]. There was a U-shaped relationship between energy cost and step width [5]. Walking with a wider step width led to greater mechanical work by lower limb muscles to redirect the COM, which influenced the energy demand [75]. As per the biomechanical and physiological responses to increased step width, it could be inferred that step width may affect the running economy. Such work reported that lower vertical GRF, lower peak medial-lateral GRF, and lower anterior-posterior GRF were economic factors [77–80]. Greater lower limb muscular activity was also associated with running economy [80]. The obvious relationship between muscular activity and running economy derived from the fact that muscles required oxygen to activate, and greater lower limb muscular activity was expected to necessitate higher oxygen consumption and lead to a lower running economy [78, 80, 81]. Based on the investigations of step width, Sample et al. [35] and Wang et al. [54] found that medial GRF and vertical GRF increased with step width, and the muscle activation of soleus, gastrocnemius, rectus femoris, vasti, gluteus maximus, gluteus medius, biceps femoris, and adductors was increased with wider steps during running [54]. Hence, changes in biomechanical and physiological parameters from wider step width could imply reduced running and walking economy [82, 83]. Future studies should focus on the strategy of adjusting step widths during sprinting to achieve higher driving force at the start and higher running economy during distance running.

### Limitations

Although the inclusion and exclusion criteria for this systematic review were strictly adhered to, there are still a few limitations that should be noted. Firstly, due to the lack of comparable data, this article did not carry out a meta-analysis of the research and data obtained. Secondly, step width was a crucial spatiotemporal variable with various internal (BMI, foot type, sex, age) and external (footwear, environment, activity level) factors that can contribute to the variations in biomechanical alteration during walking, running, and sprinting activities. Related variables have not been extensively deciphered and examined due to limited data and evidence in this review. Future research should take these potential confounding factors into account, develop well-designed experimental setups, and explore the effect of long-term changes in step width on gait biomechanics and the impact of gait retraining aiming to alter step width.

## Conclusion

In summary, short-term changes in step width during walking, running and sprinting influenced multiple measures of lower extremity biomechanics. A narrower step width may result in poor balance and higher impact loading in the lower extremities during walking and running and may limit an athlete’s sprint performance. A wider step width may be beneficial in injury management, i.e., for patients with patellofemoral pain syndrome, iliotibial band syndrome or tibial bone stress injury, to re-distribute load. Wider steps increase the supporting base and typically enhance balance control, which in turn could reduce the risks of falling during daily activities. Our synthesis of published research related to biomechanics warrants consideration in the risk reduction of lower limb injuries and in the risk of falling during locomotion. Altering the step width is proposed as a simple and non-invasive treatment method in clinical practice.

### Electronic Supplementary Material

Below is the link to the electronic supplementary material.

Supplementary Material 1

## Data Availability

The original datasets during this study are available upon request from the corresponding authors.

## Abbreviations

AP: Anterior-Posterior
COM: Center of Mass
EMG: Electromyography
GRF: Ground Reaction Force
ITBS: Iliotibial Band Syndrome
ML: Mediolateral
MOS: Margin of Stability
OA: Osteoarthritis
PFP: Patellofemoral Pain
PICO Model: Patients, Intervention, Comparator, and Outcome model
RCT: Randomized Controlled Trial
ROM: Range of Motion
STJ: Subtalar Joint
