# Supplementary material for: The Biomechanical Influence of Step Width on Typical Locomotor Activities: A Systematic Review
Source: Sports Med Open. 2024 Jul 27;10:83. doi: 10.1186/s40798-024-00750-4 (PMC11283446; doi:10.1186/s40798-024-00750-4)
Supplement: Supplementary file 1 — Supplementary Material 1 [file 40798_2024_750_MOESM1_ESM.pptx]

## Slide 1
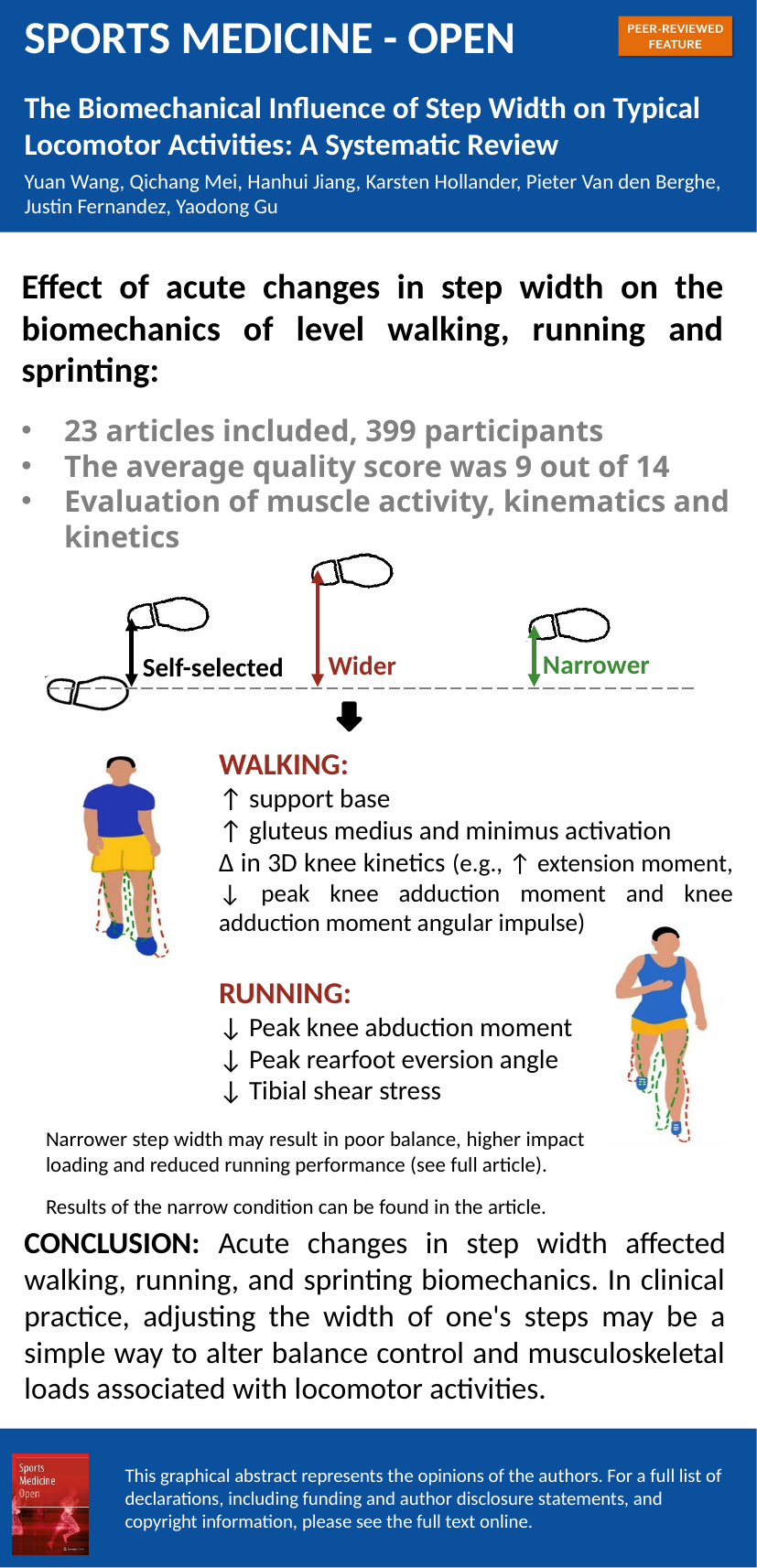

SPORTS MEDICINE - OPEN
The Biomechanical Influence of Step Width on Typical Locomotor Activities: A Systematic Review
Yuan Wang, Qichang Mei, Hanhui Jiang, Karsten Hollander, Pieter Van den Berghe, Justin Fernandez, Yaodong Gu
Effect of acute changes in step width on the biomechanics of level walking, running and sprinting:
23 articles included, 399 participants
The average quality score was 9 out of 14
Evaluation of muscle activity, kinematics and kinetics
Narrower
Wider
Self-selected
WALKING:
↑ support base
↑ gluteus medius and minimus activation
∆ in 3D knee kinetics (e.g., ↑ extension moment, ↓ peak knee adduction moment and knee adduction moment angular impulse)
RUNNING:
↓ Peak knee abduction moment
↓ Peak rearfoot eversion angle
↓ Tibial shear stress
Narrower step width may result in poor balance, higher impact loading and reduced running performance (see full article).
Results of the narrow condition can be found in the article.
CONCLUSION: Acute changes in step width affected walking, running, and sprinting biomechanics. In clinical practice, adjusting the width of one's steps may be a simple way to alter balance control and musculoskeletal loads associated with locomotor activities.
This graphical abstract represents the opinions of the authors. For a full list of declarations, including funding and author disclosure statements, and copyright information, please see the full text online.
